# Supplementary material for: A high-resolution mRNA expression time course of embryonic development in zebrafish
Source: eLife. 2017 Nov 16;6:e30860. doi: 10.7554/eLife.30860 (PMC5690287; doi:10.7554/eLife.30860)
Supplement: Supplementary file 6. [file elife-30860-supp6.zip › biolayout-clusters-files/Cluster142-genes.html]

Cluster142


# Cluster142: Genes

| Ensembl ID | Gene Name | Chr | Start | End | Biotype |
| --- | --- | --- | --- | --- | --- |
| ENSDARG00000037190 | ENSDARG00000037190 | 20 | 6164739 | 6171275 | protein\_coding |
| ENSDARG00000091699 | capn2a | 13 | 42600056 | 42630618 | protein\_coding |
| ENSDARG00000057210 | has3 | 18 | 17034374 | 17048364 | protein\_coding |
| ENSDARG00000062446 | neurl2 | 12 | 18403079 | 18405347 | protein\_coding |
| ENSDARG00000077388 | obsl1b | 9 | 41943048 | 41983030 | protein\_coding |
| ENSDARG00000044167 | padi2 | 6 | 47845001 | 47867099 | protein\_coding |
| ENSDARG00000069922 | popdc2 | 9 | 21413671 | 21420152 | protein\_coding |
| ENSDARG00000100968 | si:ch211-1a19.3 | 11 | 13572448 | 13582003 | protein\_coding |
| ENSDARG00000104587 | srl | 3 | 55900839 | 55956073 | protein\_coding |
